# Clinical significance of tumor-infiltrating lymphocytes investigated using routine H&E slides in small cell lung cancer

**DOI:** 10.1186/s13014-022-02098-z

**Published:** 2022-07-18

**Authors:** Guangrun Zhou, Jifang Zheng, Zhiwei Chen, Dan Hu, Suyu Li, Wu Zhuang, Zhiyong He, Gen Lin, Biao Wu, Wei Zhang, Weimin Fang, Fei Zheng, Jiezhong Wang, Gang Chen, Mingqiu Chen

**Affiliations:** 1 Department of Radiation Oncology, Fujian Medical University Cancer Hospital, Fujian Cancer Hospital, Fuzhou, China; 2 College of Clinical Medicine for Oncology, Fujian Medical University, Fuzhou, China; 3 Department of Radiation Oncology, Fujian Maternity and Child Health Hospital College of Clinical Medicine for Obstetrics & Gynecology and Pediatrics, Fujian Medical University, Fujian Maternity and Child Health Hospital, Fuzhou, China; 4 Fuzhou Center for Disease Control and Prevention, Fuzhou, China; 5 Department of Pathology, Fujian Medical University Cancer Hospital, Fujian Cancer Hospital, Fuzhou, China; 6 Department of Thoracic Oncology, Fujian Medical University Cancer Hospital, Fujian Cancer Hospital, Fuzhou, China; 7 Department of Thoracic Surgery, Fujian Medical University Cancer Hospital, Fujian Cancer Hospital, Fuzhou, China

**Keywords:** TILs, SCLC, Routine H&E slides, Prognosis

## Abstract

**Background:**

Tumor-infiltrating lymphocytes (TILs), investigated using routine hematoxylin and eosin (H&E)-stained section slides (H&E-sTILs), provide a robust prognostic biomarker in various types of solid cancer. The purpose of the present study was to investigate the prognostic significance of H&E-sTILs in patients with small cell lung cancer (SCLC).

**Methods:**

The clinical data of patients with SCLC who had been treated in our cancer center between January 2013 and October 2019 were collected and retrospectively reviewed. The H&E-sTILs were re-assessed by two experienced pathologists independently. H&E-sTILs that affected the overall survival (OS), progression free survival (PFS) and brain-metastasis free survival (BMFS) rates were explored using the Kaplan–Meier method, and the log-rank test was used to assess the differences. Multivariate analysis was subsequently performed using the Cox proportion hazards model.

**Results:**

A total of 159 patients with SCLC who fulfilled the inclusion criteria were enrolled in the current study. The OS rates at 1, 2 and 3 years were 59.8, 28.6 and 19.8%, respectively, for the whole group. The 3-year OS, PFS and BMFS rates for the H&E-sTILs(+) and H&E-sTILs(−) groups were 25.1% cf. 5.1% (*P* = 0.030), 14.0% cf. 4.0% (*P* = 0.013), and 66.0% cf. 11.4% (*P* = 0.023), respectively. Multivariate analyses subsequently revealed that H&E-sTILs, clinical M stage, the cycles of chemotherapy and short-term response to thoracic radiotherapy were independent factors affecting OS, whereas H&E-sTILs, clinical N stage, clinical M stage and short-term response to chemotherapy were factors affecting PFS. The H&E-sTILs affected OS, PFS and BMFS simultaneously.

**Conclusions:**

The results of this retrospective study have shown that H&E-sTILs may be considered as a prognostic biomarker affecting the short-term response to treatment, and they are the one and only risk factor for BMFS. However, due to the limitations of the nature of the retrospective design and shortcomings in visually assessing the TILs based on the H&E-stained slides, further prospective studies are required to confirm these conclusions.

## Background

Tumor-infiltrating lymphocytes (TILs), comprising T cells, B cells, and natural killer (NK) cells, have recently been shown to serve as an effective prognostic biomarker in various types of solid cancer [1–3]. Although immunohistochemistry (IHC) has been applied to differentiate the various TIL subsets, to assess their density, distribution and localization, and to evaluate their function, this method cannot be used to assess entirely the role of TILs in cancer. Using routine hematoxylin and eosin (H&E) staining to assess TILs in tissue H&E-stained section slides (H&E-sTILs) is an easy procedure that may be integrated into the workflow of pathology laboratories without extra staining protocols, and which has been shown to be a valuable prognostic biomarker of patients [2, 4–9]. However, to date, no consensus has been reached on a standard method of quantification for H&E-sTILs in small cell lung cancer (SCLC), and neither has the manner in which H&E-sTILs affect SCLC been identified, especially with respect to predicting brain metastasis (BM) [10–12]. The aim of the present study was therefore to assess the value of H&E-sTILs in patients with SCLC.

## Methods

### Patient selection and data collection

The present retrospective study was approved by the Fujian Province Cancer Hospital Institutional Review Board. The eligibility and exclusion criteria employed in the current study were similar to those of a previously published study [13]. In brief, the eligibility criteria were as follows: primary histologically proven SCLC; a sufficiently good performance status to enable treatment; efficient pretreatment workup for tumor staging and treatment response evaluation; complete follow-up data; good quality of H&E slides and/or adequate tissue in paraffin-embedded formalin-fixed blocks; and treatment with at least one cycle of chemotherapy of a dual-agent. Patients who survived for < 1 month following treatment were considered as adverse event fatalities, and were excluded from the present study.

### Treatment strategy

All patients in the current study were administered chemotherapy with at least one cycle of a dual-agent combination of etoposide or irinotecan with cisplatin (EP, IP), carboplatin (EC), lobaplatin (EL) or nidaplatin (EN). Thoracic radiotherapy (TRT) with a ≥ 45 Gy radiation dose was performed using the intensity-modulated radiotherapy (IMRT) technique when necessary, depending on the clinician and the patient’s condition. The details of TRT, including the gross tumor volume (GTV), clinical tumor volume (CTV) and organ at risk (OAR), have been reported previously [13].

### Criteria for treatment toxicity and the short-term response

The toxicities of chemotherapy or TRT were evaluated according to the National Cancer Institute Common Toxicity Criteria (NCI CTC) v.4.0 [14] or the Radiation Therapy Oncology Group (RTOG) criteria [15], respectively.

The short-term response of chemotherapy or TRT was evaluated at 3–4 weeks after the most recent cycle of chemotherapy or the completion of TRT, and subsequently confirmed 4 weeks later. The short-term responses were categorized as a clinically complete response (CR), a partial response (PR), stable disease (SD) or progressive disease (PD) according to the guidelines of RECIST1.1 [16]. The CR and PR categories were considered as the sensitive-to-treatment group, whereas the SD and PD were considered as the resistance-to-treatment group in the current study.

### TIL assessment

Histopathological assessment of TILs was performed on H&E slides by two experienced pathologists (Dan Hu and Gang Chen) according to the International Immuno-Oncology Biomarkers Working Group [17]. Briefly, every patient was assessed with regard to TILs in at least two section (4–5 µm) by microscopy (magnification of ×200–400; ocular magnification, ×10, objective magnification, ×20–×40). The tissue was primarily obtained from primary biopsies, although some were from lymph node biopsies. The primary method for evaluating sTIL in lymph nodes was based on partial metastatic tumor deposits, which can be determined under a microscope, and the boundary between pre-existing lymphatic tissue and tumor sTIL was clearly discernible. The pre-existing lymphoid stroma was excluded from the evaluation. To minimize selection bias, at least four standard-compliant vision fields per section were randomly selected, and the mean infiltrative areas were considered as the last results enrolled for analysis.

**Fig. 1:**
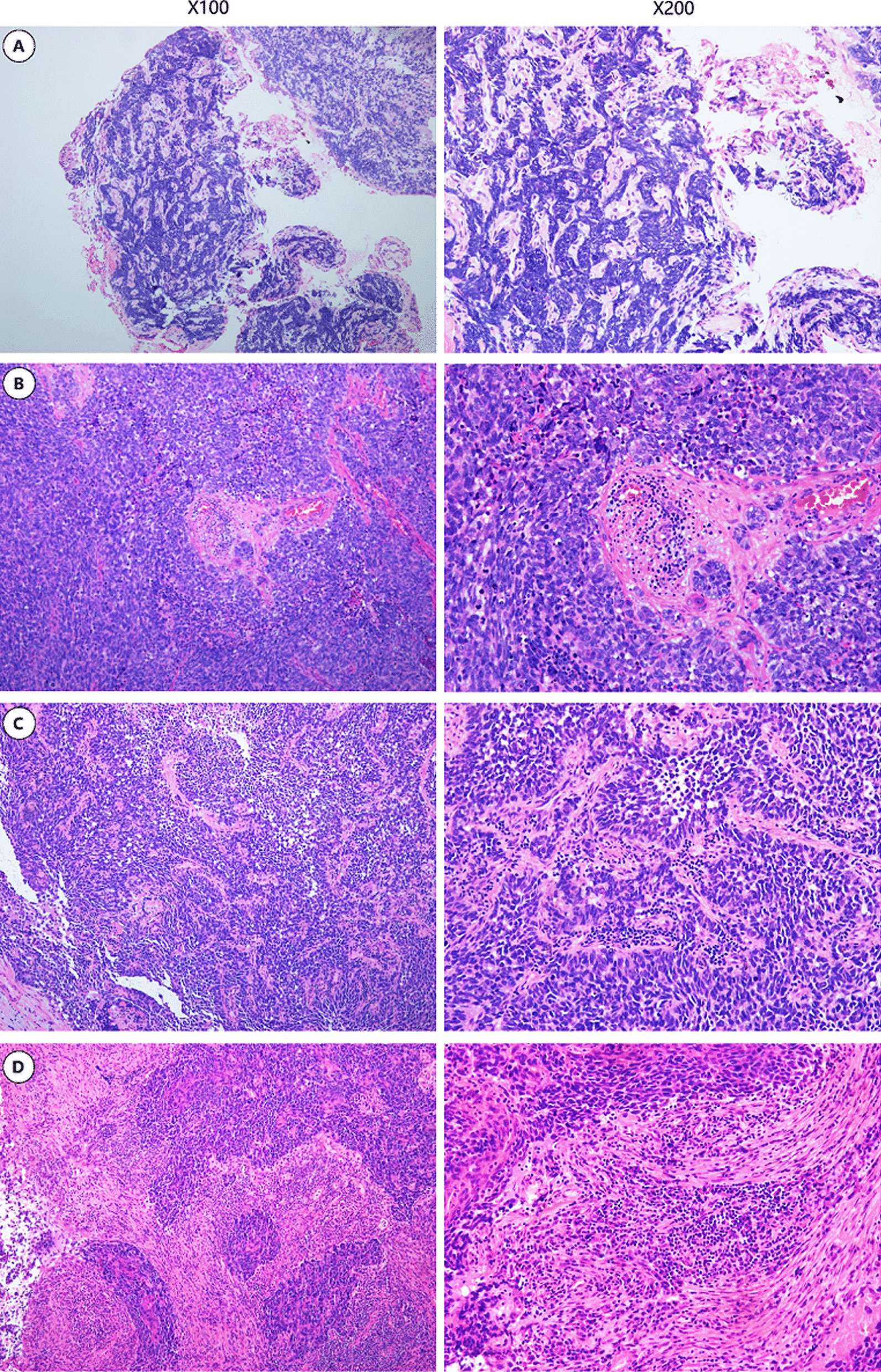
Percentage level of sTIL on H&E stained sections. Infiltration ratio** A** <10%,** B** 10–20%,** C** 21–50%,** D** >50% (100X magnification for the left column and 200x magnification for the right column)

Considering that our pre-study TIL assessment indicated that intratumoral TILs were rarely found, we used the stromal TIL assessment of H&E staining [18] in the current study (Fig. 1). The denominator used to determine the percentage of stromal TILs was the area of stromal tissue (i.e. the area occupied by mononuclear inflammatory cells over the total intratumoral stromal area), not the number of stromal cells (i.e. the fraction of total stromal nuclei that represents mononuclear inflammatory cell nuclei) [17]. However, due to the limitation of standard values and the lower numbers of H&E-sTILs identified in SCLC to date, the H&E-sTILs status was defined simply as either positive(+) or negative (−) for subsequent analysis as follows: ≤ 0%, negative (−), > 1%, positive (+); similar to the method of analysis used by Rao et al. [19].

### Surveillance and statistical analysis

The survival outcomes were evaluated in June 2020. The outcomes of interest were the overall survival (OS), progression-free survival (PFS), BM-free survival (BMFS) and BM rates. The survival time was calculated in a similar manner to that described in our previous study [13]. In brief, OS was defined from the date of diagnosis to either the date of death or the date of the last follow-up. The PFS was calculated from the date of diagnosis to the date of disease progression, whereas the BMFS was determined as the duration between the date of diagnosis and BM. Patients who were censored at the last follow-up date or who had died without evidence of BM were censored for BM incidence [13].

Data were analyzed using SPSS version 24.0 (IBM Corp., Armonk, NY, USA). The survival curves were constructed using the Kaplan–Meier method, and comparisons were performed using log-rank tests. Univariate and multivariate analyses of the associations of clinical baseline characteristics [including sex, age, H&E-sTILs, clinical TNM (cTNM) stage including clinical T stage (cT), clinical N stage (cN) and clinical M stage (cM), prophylactic cerebral irradiation (PCI), regimens and cycles of chemotherapy, TRT dose and short-term response to chemotherapy or TRT] with OS, PFS and BMFS rates were performed using the Cox proportional hazards model. Confidence intervals (CIs) represented 95% lower and upper limits. The variables that were statistically significantly correlated with OS, PFS or BMFS were entered in the multivariate analysis using logistic regression.

Receiver operating characteristic (ROC) curve analysis was applied to establish the cut-off values of continuous variables using the area under the curve (AUC). Finally, propensity score matching (PSM) analyses were used to minimize the differences in characteristics between the compared groups, similar to the procedure followed in our previous study [20]. In brief, all the imbalances in tumor variables that may have affected the OS were compared using a χ^2^ test first. Then, a propensity score was calculated using the variables that were statistically significantly correlated with OS in the logistic regression analysis in the multivariate analysis. Finally, all analyses regarding OS were adjusted based on the generated propensity score. A Pearson’s χ^2^ test was subsequently performed to compare the differences between the H&E-sTILs (−) and H&E-sTILs (+) groups after matching.

## Results

### Patient characteristics

Between February 2012 and August 2018, 173 patients were reviewed. A total of 159 patients who fulfilled the inclusion criteria were enrolled in the current study, of whom 77 (48.4%) patients were determined to be H&E-sTILs(+) and 82 (51.6%) were H&E-sTILs(−). The differences in clinical characteristics, including sex, age, cTNM stage, regimens and cycles of chemotherapy, TRT, dose of TRT and PCI between the H&E-sTILs(+) and H&E-sTILs(−) patient groups were not significant (Table 1).

**Table 1:** Clinical characteristics of patients

	Total	sTIL+	sTIL−	*p*	
Gender				0.343	
Male	145	69	76		
Female	14	8	6		
Median age (year, rang)	59.5 (24–84)	58.8 (38–84)	60.0 (24–77)	0.624	
Clinical T stage				0.250	
T1	16	10	6		
T2	39	16	23		
T3	52	21	31		
T4	52	30	22		
Mean Dmax-T (cm)	5.613	5.56	5.66	0.672	
Clinical N stage				0.520	
0	13	6	7		
1	6	4	2		
2	74	33	41		
3	66	34	32		
Clinical M stage				0.270	M0 versus M1
M0	90	46	44		
M1	69	31	38		
EM only	51	24	27	0.587	EM versus BM
BM only	8	4	4		
EM and BM	10	3	7		
Clinical TNM stage				0.301	
I	1	1	0		
II	4	1	3		
III	86	44	42		
IV	68	31	37		
Regimen of CT				0.539	
EP	107	50	57		
EC	34	15	19		
EN	6	4	2		
IP	6	4	2		
Others	6	4	2		
Median cycles of CT (range)	4.7 (1–8)	4.7 (1–8)	4.7 (1–7)	0.870	
TRT				0.173	
No	77	33	44		
Yes	82	44	38		
Median dose of TRT (cGy, range)	5599 (3600–6900)	5595 (40,006–900)	5604 (3600–6400)	0.682	
PCI				0.414	
No	151	72	79		
Yes	8	5	3		

BM, brain metastasis; CT, chemotherapy; Dmax-T, greatest dimension of tumor; EP, etoposide with cisplatin; EC, etoposide with carboplatin; EM, extracranial metastasis; EN, etoposide with nidaplatin; IP, irinotecan with cisplatin; TRT, thoracic radiotherapy

### Short-term response to treatment and H&E-sTILs

The short-term response to chemotherapy and TRT values are presented in Table 2. Irrespective of whether the chemotherapy was administered alone or as TRT combined with chemotherapy, patients who were H&E-sTILs(+) exhibited sensitivity in terms of the short-term response to the treatment, whereas patients who were H&E-sTILs(−) displayed resistance in terms of their short-term responses to treatment.

**Table 2:** Results of treatment

	Total	sTIL+	sTIL−	*p*
Response to chemotherapy (case)				0.030
Treatment Sensitive (CR + PR)	123	65	58	
Treatment Resistant (SD + PD)	36	12	24	
Response to TRT (case)*				0.045
Treatment Sensitive (CR + PR)	63	38	26	
Treatment Resistant (SD + PD)	18	6	12	
mOS (moths)	16	18	12	0.030
1, 2, 3-year OS rate	59.8%, 28.6%, 19.8%	72.2%, 37.5%, 25.1%	48.0%, 19.9%, 5.1%	0.030
1, 2, 3-year PFS rate	31.5%, 13.1%, 6.2%	37.2%, 21.0%, 14.0%	25.9%, 4.2%, 4.2%	0.013
1, 2, 3-year BMFS rate**	75.0%, 47.2%, 47.2%	75.1%, 66.0%, 66.0%	75.5%, 11.4%, 11.4%	0.023

*Patients administratived TRT and chemotherapy

**Patients with non-BM and non-PCI

### OS, PFS and TILs

The median follow-up time in the entire cohort and in the surviving patients was 16 (range: 2–62) and 19 (range: 6–62) months, respectively. At the last follow-up, 48 patients remained alive and 111 patients had died, of whom 48 patients (48/111; 43.2%) had succumbed to extracranial progression (including locoregional or distant recurrence) alone, 20 to BM, 8 to both, and 35 to unascertainable intracranial or/and extracranial progression; those 35 patients were considered to have died from unknown causes when conducting the survival analysis.

**Fig. 2:**
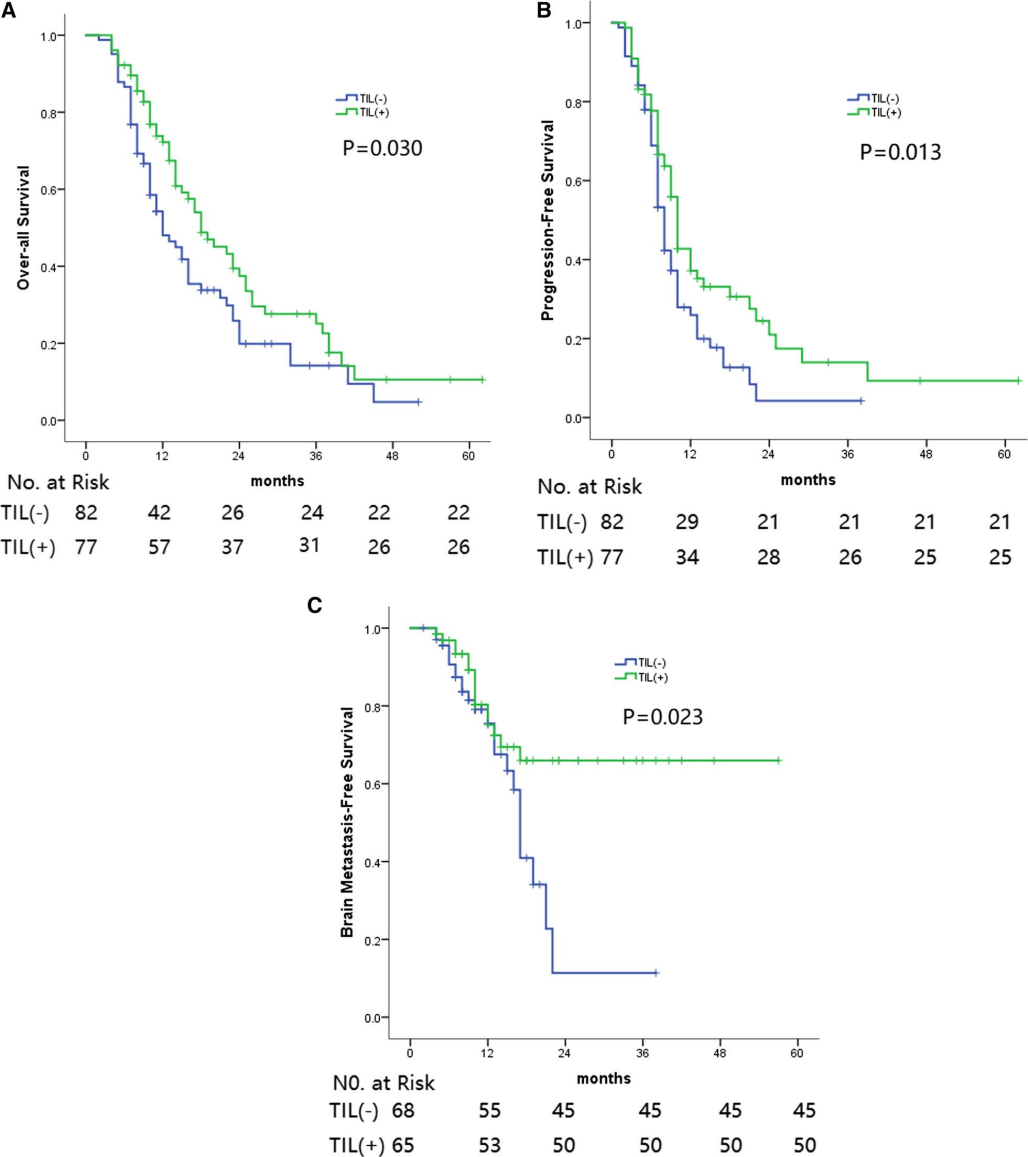
**A** Overall Survival between TIL(-) and TIL(+) in the whole cohort.** B** Progression-Free Survival between TIL(-) and TIL(+) in the whole cohort.** C** Brain Metastasis-Free Survival between TIL(-) and TIL(+) in the non-PCI and non-BM patients

The OS and PFS rates at the 1-, 2- and 3-year stages for the entire group, the H&E-sTILs(+) group and the H&E-sTILs(−) group are summarized in Table 2. The mOS of patients with H&E-sTILs(+) was markedly superior compared with patients with H&E-sTILs(−) (18 months cf. 12 months; *P* = 0.030) (Fig. 2A). The difference in mPFS between the two groups was also found to be statistically significant (10 months cf. 8 months, *P* = 0.013) (Fig. 2B).

Univariate and multivariate analyses for the entire group revealed that H&E-sTILs, cM stage, the cycles of chemotherapy and short-term response to TRT were independent factors affecting OS, whereas H&E-sTILs, cN stage, cM stage and short-term response to chemotherapy were considered as factors affecting PFS (Table 3).

**Table 3:** Prognostic factors by univariate and multivariate analyses for OS, PFS and BMFS

	OS						PFS						BMFS (in non-BM and non-PCI)		
	Univariate analyses			Multivariate analyses			Univariate analyses			Multivariate analyses			Univariate analyses		
	*P*	HR	(95% CI)	*P*	HR	(95% CI)	*P*	HR	(95% CI)	*P*	HR	(95% CI)	*P*	HR	(95% CI)
Gender	0.542	1.226	0.638–2.355				0.754	0.896	0.452–1.776				0.140	0.224	0.031–1.631
Age	0.867	1.002	0.980–1.025				0.525	0.993	0.972–1.015				0.519	0.988	0.954–1.024
sTIL	0.041	0.675	0.463–0.984	0.027	0.648	0.441–0.951	0.020	0.637	0.436–0.931	0.019	0.632	0.430–0.927	0.029	0.477	0.245–0.927
Clinical T stage	0.288	0.896	0.732–1.097				0.293	1.112	0.912–1.355				0.995	0.999	0.726–1.375
Dmax-T	0.227	1.046	0.973–1.125				0.016	1.087	1.016–1.163				0.783	1.017	0.904–1.142
Clinical N stage	0.014	1.355	1.064–1.724				0.012	1.347	1.069–1.699	0.027	1.298	1.030–1.635	0.218	1.292	0.860–1.940
Clinical M stage	0.000	2.153	1.469–3.156	0.025	1.695	1.069–2.688	0.000	2.128	1.436–3.153	0.001	1.960	1.306–2.941	0.572	0.810	0.390–1.683
Clinical TNM stage	0.001	1.901	1.315–2.749				0.000	1.984	1.371–2.869				0.595	0.845	0.455–1.570
Regimen of CT	0.582	0.951	0.794–1.138				0.158	1.121	0.956–1.315				0.519	0.903	0.663–1.230
Cycles of CT	0.007	0.831	0.727–0.950	0.019	0.835	0.718–0.971	0.352	0.939	0.822–1.072				0.229	1.168	0.907–1.503
TRT	0.000	0.294	0.195–0.445				0.000	0.493	0.333–0.730				0.370	1.390	0.676–2.855
Dose of TRT	0.085	1.000	0.999–1.000				0.412	1.000	1.000–1.001				0.421	1.000	1.000–1.001
PCI	0.052	0.408	0.165–1.008				0.029	0.361	0.145–0.900				NA		
Response to CT	0.003	1.736	1.203–2.507				0.004	1.788	1.209–2.647	0.040	1.514	1.018–2.25	0.605	0.797	0.337–1.886
Response to TRT	0.000	1.176	1.115–1.240	0.000	1.123	1.055–1.196	0.000	1.100	1.046–1.157				0.470	0.967	0.883–1.059

**Fig. 3:**
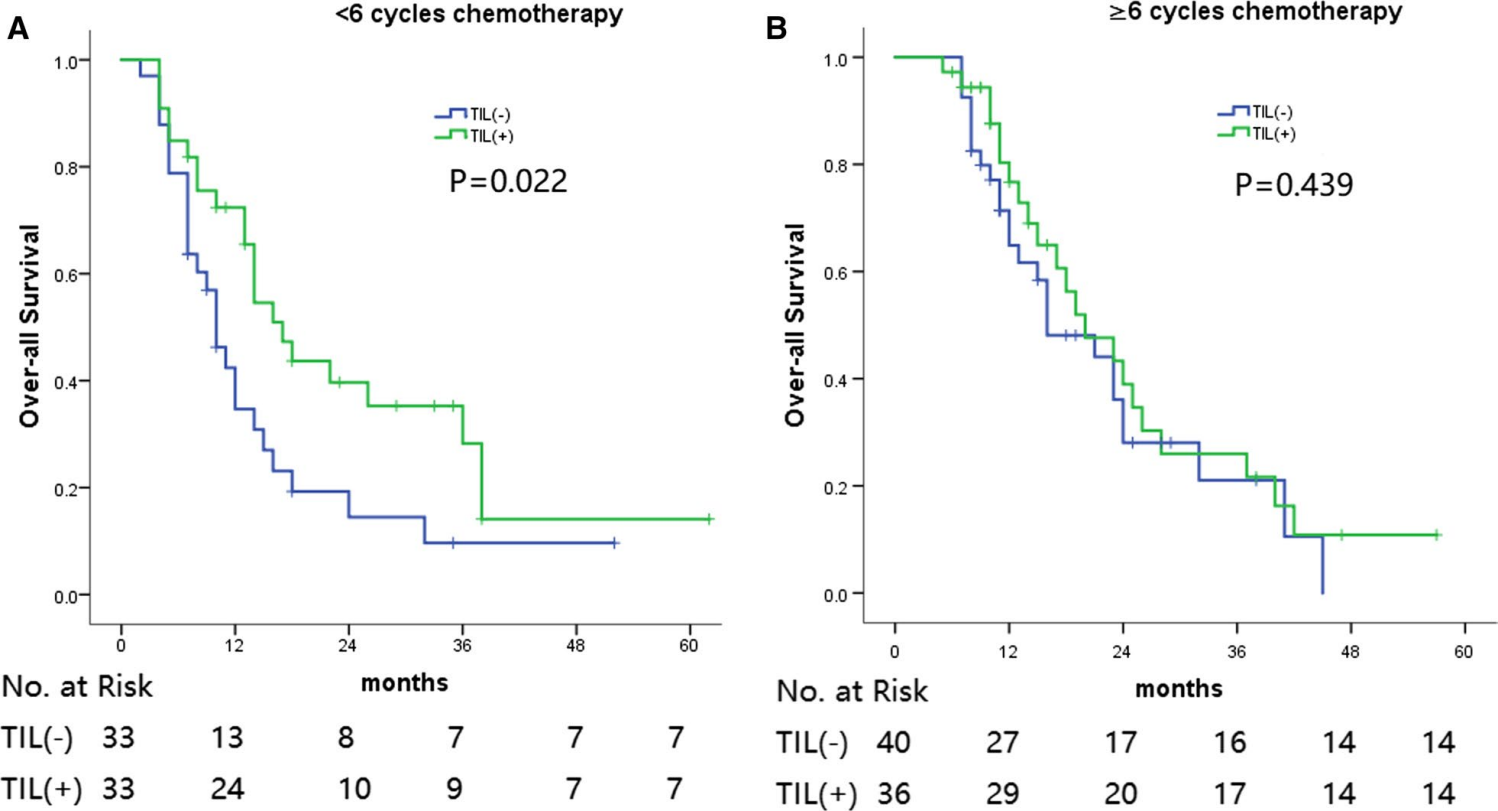
**A** Overall Survival between TIL(-) and TIL(+) in the patients administrated with <6 cycles chemotherapy.** B** Overall Survival between TIL(-) and TIL(+) in the patients administrated with ≥6 cycles chemotherapy

**Fig. 4:**
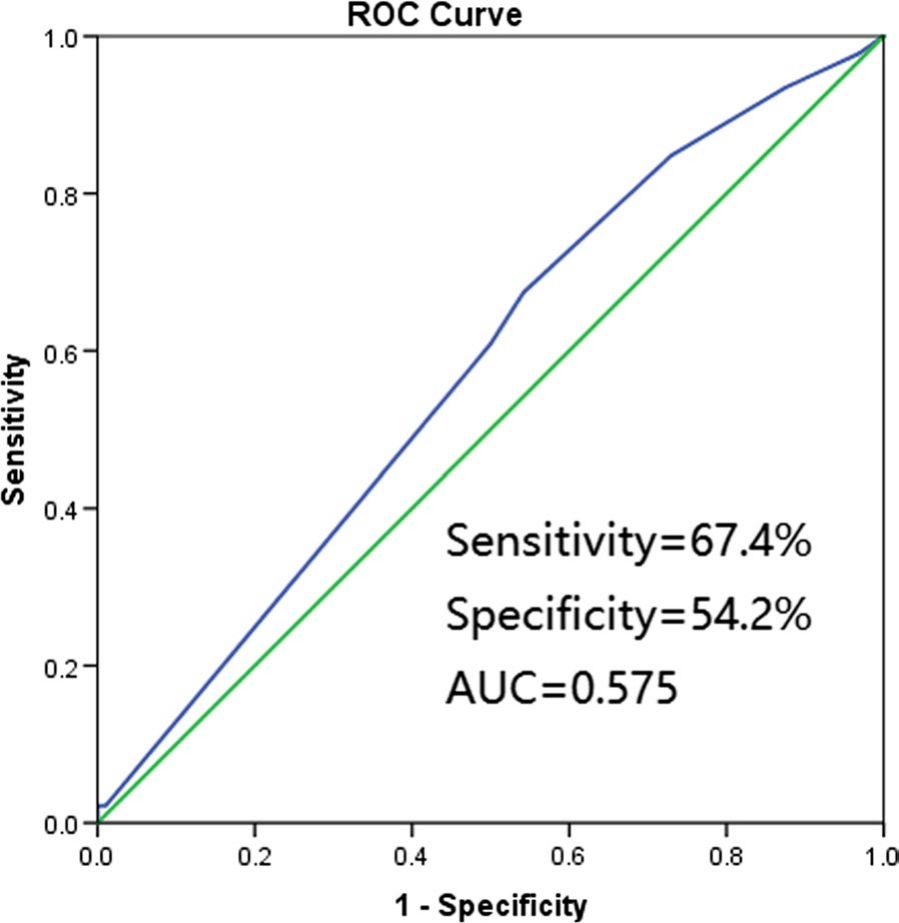
ROC to predict chemotherapy cycles. AUC was 0.575. AUC, area under the curve

As far as the entire cohort of patients was concerned, patients who received ≥ 6 cycles of chemotherapy achieved markedly improved OS rates compared with patients with < 6 cycles chemotherapy. However, following PSM analysis, the difference was found to be not significant. Furthermore, in the PSM subgroups, the difference between the OS rates of the H&E-sTILs(−) and H&E-sTILs(+) groups was significant in patients who received < 6 cycles chemotherapy, but not with patients who received ≥ 6 cycles chemotherapy (Figs. 3A, B, 4).

### BM, BMFS and TILs analysis

The correlation between H&E-sTILs and BM was subsequently explored in the ‘non-PCI and non-BM at initial diagnosis’ subgroup (Table 2). A total of 133 patients were enrolled in the subgroup analysis, of whom 38 (38/133; 28.6%) patients in the subgroup experienced BM post-treatment. The 1-, 2- and 3-year BMFS rates of patients in the H&E-sTILs(−) and H&E-sTILs(+) groups are summarized in Table 2 (Fig. 2C). Univariate and multivariate analyses indicated that the H&E-sTILs were the unique factor affecting the BMFS (Table 3).

### TILs and cTNM stage analysis

**Fig. 5:**
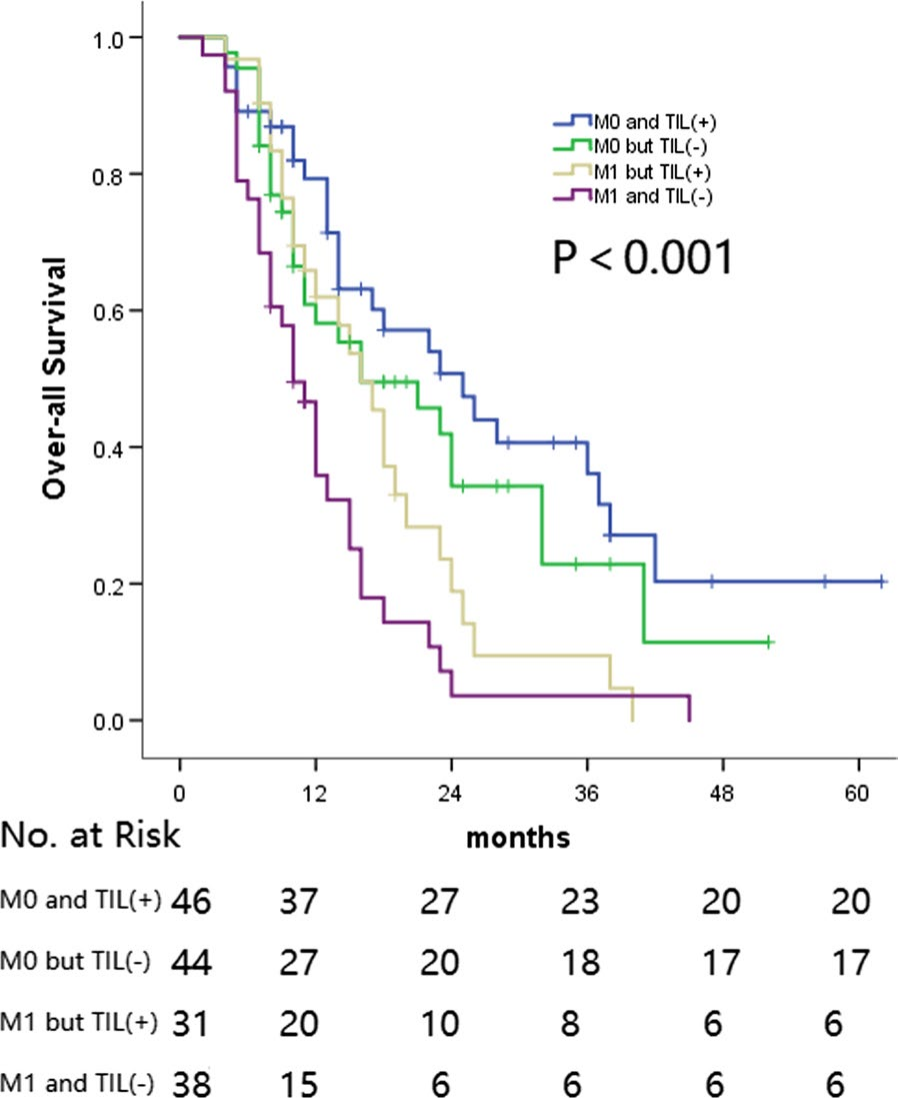
Overall survival of patients according to the different combination of M stage and TIL

The cM stage is considered as one independent significant risk factor according to the univariate and multivariate analyses explored in the entire group. For the entire group, patients with M0 stage cancer were shown to have increased prospects of survival compared with patients with M1 stage cancer. Comparing the survival analyses of the M0 and H&E-sTILs(+), M0 and H&E-sTILs(−), M1 and H&E-sTILs(+) and M1 and H&E-sTILs(−) subgroups revealed a sequentially decreasing survival rate. The M0 and H&E-sTILs(+) group was associated with the best prospect of survival, whereas the M1 and H&E-sTILs(−) subgroup had the worst survival outlook; in addition, the difference in survival rate between the M0 and H&E-sTILs(−) and M1 and H&E-sTILs(+) subgroups was found to be not significant (Fig. 5).

## Discussion

TILs have been demonstrated to serve as a prognostic factor in several types of solid cancer [2, 4–9], although studies on TILs in SCLC have only been rarely reported. A couple of decades ago, Eerola et al. [10] performed a study to analyze the associations of T cells using IHC in the prognosis of patients with operated SCLC. This group found that higher levels of TILs were associated with significantly more favorable survival times. However, it is not clear whether all mononuclear immune cells are T cells, and the relative proportions of macrophages, dendritic cells, myeloid-derived suppressor cells and plasma cells in the immune infiltrate may also serve important immune roles in cancer [21]. Furthermore, due to inaccurate measurement of the test variable without controlled calibration, the digital quantification of IHC-stained sections may yield different results and conclusions [9]. In more recent times, the histological evaluation of TILs is emerging as a more promising biomarker in various types of solid tumors [22], and this method has been proposed as a biomarker for inclusion in routine histopathological reporting and in the implementation of TNM staging for predicting patients’ prognosis [23, 24]. H&E-stained sections are easy to distinguish; thus, the evaluation of TILs using H&E-stained sections may be useful for predicting the prognosis in SCLC patients.

However, to the best of our knowledge, no similar studies have been performed to evaluate the relationships of TILs based on H&E-stained slides in SCLC. Therefore, we consider that the present study is the first to have explored H&E-sTILs in SCLC. The current study demonstrated that, for the entire cohort of enrolled patients, even in the absence of any significant differences in clinical characteristics comparing between the H&E-sTILs(+) and H&E-sTILs(−) patient groups, patients in the H&E-sTILs(+) group exhibited superior survival rates in terms of the OS, PFS and BMFS rates compared with patients in the H&E-sTILs(−) group. These results suggested that H&E-sTILs may serve as a potential biomarker in predicting prognosis of SCLC.

The short-term response to treatment, including chemotherapy or radiotherapy, is a factor that has been confirmed to be associated with survival. Liu et al.[25] reported that there is a correlation between TILs and the chemotherapy response in non-small cell lung cancer. Similarly, in the current study, irrespective of whether the patients were treated with chemotherapy alone or chemotherapy combined with TRT, those in the H&E-sTILs(+) group exhibited a greater sensitivity in terms of the short-term response to treatment compared with patients in the H&E-sTILs(−) group, which suggested that, for patients in the H&E-sTILs(−) group, more intensive treatment strategies should be implemented in order to improve their treatment response and survival prospects.

Chemotherapy is a cornerstone in the treatment of patients with SCLC [26]. In the current study, ROC analysis indicated that patients who received ≥ 6 cycles of chemotherapy achieved significantly improved OS rates compared with patients who received < 6 cycles of chemotherapy. Further analysis of the H&E-sTILs in the different subgroups of patients treated with differing numbers of chemotherapy cycles indicated that the difference in the OS rate between the H&E-sTILs(−) and the H&E-sTILs(+) subgroups was significant in patients who received < 6 cycles of chemotherapy, but for those patients who received ≥ 6 cycles of chemotherapy, the difference was not significant. These findings suggested that, for the H&E-sTILs(+) patients, a rational decision may be taken to administer fewer cycles of chemotherapy, but for the H&E-sTILs(−) patients, who were experiencing a poorer immune tumor environment and more dismal prospects of survival, more intensive treatment strategies [for example, with a greater number of cycles of chemotherapy or with the inclusion of added immune checkpoint inhibitors (ICIs)] should be considered in the clinic.

It has been shown in numerous studies that TRT is able to improve patients’ survival across the board, whether the patients have localized or extensive stage disease [26]. Similarly, the present study indicated that, compared with the non-TRT treatment group, patients who received TRT successfully achieved improved survival rates, regardless of the cTNM stage. In addition, the differences in the OS rate comparing between H&E-sTILs(−) and H&E-sTILs(+) patients in the TRT subgroup almost reached the level of statistical significance, although the failure to do so may have been influenced by the limited number of cases of enrolled patients. These results suggested that, for patients with H&E-sTILs(+), it may be possible to take a rational decision to use a lower radiation dose or a restricted field of TRT, especially when considering the lung toxicity that is associated with radiotherapy combined with ICIs.

The TNM staging system is the system accepted worldwide in terms of guiding cancer treatment and predicting prognosis, of which the M stage is associated with the most dismal prospects of survival. In the current study, neither the cT stage nor the cN stage, but only the cM stage was considered as a risk factor in the TNM staging system influencing the OS, PFS and BMFS rates. The different cM stage subgroups comprising the different H&E-sTILs statuses were subsequently explored to identify the best method for predicting patients` survival. The survival rates were found to decrease sequentially and significantly comparing among the M0 and H&E-sTILs(+), M0 and H&E-sTILs(−), M1 and H&E-sTILs(+) and M1 and H&E-sTILs(−) subgroups, although the difference between the M0 and H&E-sTILs(−) and M1 and H&E-sTILs(+) subgroups was not found to be statistically significant on account of the limited number of enrolled patients. These results demonstrated that H&E-sTILs should be considered in the TNM staging system as a prognostic factor to predict survival more accurately [23, 27].

With the development of systematic treatment strategies, especially in the case of ICIs applied in the clinic, BM has become the crucial failure model of treatment in SCLC. A study published previously by our research group [13] revealed that approximately one-third of patients encountered BM post-treatment, and poor short-term response to TRT and larger Dmax-T values were identified as risk factors for BM. However, in the current study, univariate and multivariate analyses indicated that H&E-sTILs were the unique factor affecting the BM and BMFS parameters. Although the BM rate between the H&E-sTILs(−) and H&E-sTILs(+) groups was not statistically different due to the limited number of enrolled cases and surveillance time, the difference in BMFS values exhibited a marked significance, which indicated that, for non-BM patients who were H&E-sTILs(+) at the initial diagnosis, PCI may not be necessary.

The global familiar studies Impower133 [28] and CASPIAN [29] have confirmed that ICIs achieve sustained improvements in the OS rates of patients with SCLC at the advanced and extensive stages of the disease. However, an effective biomarker of ICIs in SCLC had not yet been established, in spite of the fact that several other studies have reported that TILs may serve as a biomarker of ICI treatment [30]. Regrettably, given the fact that none of the patients enrolled in the current study received ICIs, it was not possible for us to investigate this further in terms of assessing the value of H&E-sTILs in SCLC for patients treated with ICIs, although will be an important focus of our future work.

## Conclusions

In conclusion, the present study has shown that H&E-sTILs are the one and only significant prognostic factor affecting OS, PFS and BMFS simultaneously. Compared with patients in the H&E-sTILs(+) subgroup, patients in the H&E-sTILs(−) group had shorter survival times, more frequently occurring BM, and therefore these patients should be administered more intensive treatments in the clinic.

However, the present study did have several limitations, including its retrospective design from a single institution, the variability associated with visual TIL assessments using small biopsy tissue, and both the insufficient follow-up duration of the patients and the limited number of patients enrolled. The results of our investigation must therefore be interpreted with caution, and further prospective clinical trials are required to confirm the conclusions.

## Data Availability

All datasets presented in this study are included in the article.

## Abbreviations

BM: Brain metastasis
BMFS: Brain-metastasis free survival
CR: Complete response
CTV: Clinical tumor volume
GTV: Gross tumor volume
H&E: Hematoxylin and eosin
H&E-sTILs: Assess TILs in tissue H&E-stained section slides
ICIs: Immune checkpoint inhibitors
IMRT: Intensity-modulated radiotherapy
OAR: Organ at risk
OS: Overall survival
PCI: Prophylactic cerebral irradiation
PD: Progressive disease
PFS: Progression free survival
PR: Partial response
PSM: Propensity score matching
ROC: Receiver operating characteristic
SCLC: Small cell lung cancer
SD: Stable disease
TRT: Thoracic radiotherapy
TILs: Tumor-infiltrating lymphocytes

## References

[1] Saito T, Nishikawa H, Wada H, Nagano Y, Sugiyama D, Atarashi K, Maeda Y, Hamaguchi M, Ohkura N, Sato E, Nagase H, Nishimura J, Yamamoto H, Takiguchi S, Tanoue T, Suda W, Morita H, Hattori M, Honda K, Mori M, Doki Y, Sakaguchi S. Two FOXP3(+)CD4(+) T cell subpopulations distinctly control the prognosis of colorectal cancers. Nat Med. 2016;22:679–84. 27111280 10.1038/nm.4086

[2] Brambilla E, Le Teuff G, Marguet S, Lantuejoul S, Dunant A, Graziano S, Pirker R, Douillard JY, Le Chevalier T, Filipits M, Rosell R, Kratzke R, Popper H, Soria JC, Shepherd FA, Seymour L, Tsao MS. Prognostic effect of tumor lymphocytic infiltration in resectable non-small-cell lung cancer. J Clin Oncol Off J Am Soc Clin Oncol. 2016;34:1223–30. 10.1200/JCO.2015.63.0970 PMC4872323 26834066

[3] Perez EA, Ballman KV, Tenner KS, Thompson EA, Badve SS, Bailey H, Baehner FL. Association of stromal tumor-infiltrating lymphocytes with recurrence-free survival in the N9831 adjuvant trial in patients with early-stage HER2-positive breast cancer. JAMA Oncol. 2016;2:56–64. 26469139 10.1001/jamaoncol.2015.3239 PMC4713247

[4] Rakaee M, Kilvaer TK, Dalen SM, Richardsen E, Paulsen E-E, Hald SM, Al-Saad S, Andersen S, Donnem T, Bremnes RM. Evaluation of tumor-infiltrating lymphocytes using routine H&E slides predicts patient survival in resected non-small cell lung cancer. Hum Pathol. 2018;79:188–98. 29885403 10.1016/j.humpath.2018.05.017

[5] Ruffini E, Asioli S, Filosso PL, Lyberis P, Bruna MC, Macrì L, Daniele L, Oliaro A. Clinical significance of tumor-infiltrating lymphocytes in lung neoplasms. Ann Thorac Surg. 2009;87:365–72. 19161739 10.1016/j.athoracsur.2008.10.067

[6] Horne ZD, Jack R, Gray ZT, Siegfried JM, Wilson DO, Yousem SA, Nason KS, Landreneau RJ, Luketich JD, Schuchert MJ. Increased levels of tumor-infiltrating lymphocytes are associated with improved recurrence-free survival in stage 1A non-small-cell lung cancer. J Surg Res. 2011;171:1–5. 21571304 10.1016/j.jss.2011.03.068

[7] Kilic A, Landreneau RJ, Luketich JD, Pennathur A, Schuchert MJ. Density of tumor-infiltrating lymphocytes correlates with disease recurrence and survival in patients with large non-small-cell lung cancer tumors. J Surg Res. 2011;167:207–10. 19896677 10.1016/j.jss.2009.08.029

[8] Feng W, Li Y, Shen L, Cai X-W, Zhu Z-F, Chang J-H, Xiang J-Q, Zhang Y-W, Chen H-Q, Fu X-L. Prognostic value of tumor-infiltrating lymphocytes for patients with completely resected stage IIIA (N2) non-small cell lung cancer. Oncotarget. 2016;7:7227. 26811495 10.18632/oncotarget.6979 PMC4872781

[9] Hendry S, Salgado R, Gevaert T, Russell PA, John T, Thapa B, Christie M, van de Vijver K, Estrada MV, Gonzalez-Ericsson PI, Sanders M, Solomon B, Solinas C, Van den Eynden G, Allory Y, Preusser M, Hainfellner J, Pruneri G, Vingiani A, Demaria S, Symmans F, Nuciforo P, Comerma L, Thompson EA, Lakhani S, Kim SR, Schnitt S, Colpaert C, Sotiriou C, Scherer SJ, Ignatiadis M, Badve S, Pierce RH, Viale G, Sirtaine N, Penault-Llorca F, Sugie T, Fineberg S, Paik S, Srinivasan A, Richardson A, Wang Y, Chmielik E, Brock J, Johnson DB, Balko J, Wienert S, Bossuyt V, Michiels S, Ternes N, Burchardi N, Luen SJ, Savas P, Klauschen F, Watson PH, Nelson BH, Criscitiello C, O’Toole S, Larsimont D, de Wind R, Curigliano G, Andre F, Lacroix-Triki M, van de Vijver M, Rojo F, Floris G, Bedri S, Sparano J, Rimm D, Nielsen T, Kos Z, Hewitt S, Singh B, Farshid G, Loibl S, Allison KH, Tung N, Adams S, Willard-Gallo K, Horlings HM, Gandhi L, Moreira A, Hirsch F, Dieci MV, Urbanowicz M, Brcic I, Korski K, Gaire F, Koeppen H, Lo A, Giltnane J, Rebelatto MC, Steele KE, Zha J, Emancipator K, Juco JW, Denkert C, Reis-Filho J, Loi S, Fox SB. Assessing tumor-infiltrating lymphocytes in solid tumors: a practical review for pathologists and proposal for a standardized method from the international immunooncology biomarkers working group: part 1: assessing the host immune response, TILs in invasive breast carcinoma and ductal carcinoma in situ, metastatic tumor deposits and areas for further research. Adv Anat Pathol. 2017;24:235–51. 28777142 10.1097/PAP.0000000000000162 PMC5564448

[10] Eerola A, Soini Y, Pääkkö P. A high number of tumor-infiltrating lymphocytes are associated with a small tumor size, low tumor stage, and a favorable prognosis in operated small cell lung carcinoma. Clin Cancer Res Off J Am Assoc Cancer Res. 2000;6:1875–81. 10815910

[11] Wang W, Hodkinson P, McLaren F, Mackean M, Williams L, Howie S, Wallace W, Sethi T. Histologic assessment of tumor-associated CD45(+) cell numbers is an independent predictor of prognosis in small cell lung cancer. Chest. 2013;143:146–51. 22847040 10.1378/chest.12-0681

[12] Bonanno L, Pavan A, Dieci M, Di Liso E, Schiavon M, Comacchio G, Attili I, Pasello G, Calabrese F, Rea F, Favaretto A, Rugge M, Guarneri V, Fassan M, Conte P. The role of immune microenvironment in small-cell lung cancer: distribution of PD-L1 expression and prognostic role of FOXP3-positive tumour infiltrating lymphocytes. Eur J Cancer (Oxford, England: 1990). 2018;101:191–200. 10.1016/j.ejca.2018.06.023 30077124

[13] Wu S, Wang J, Zhang W, Li J, Wu H, Huang Z, Zhou G, Pan J, Chen M. Analysis of factors affecting brain metastasis in limited-stage small-cell lung cancer treated with definitive thoracic irradiation. Front Oncol. 2020;10:556634. 33194620 10.3389/fonc.2020.556634 PMC7658601

[14] U.D.o. Health, N.C.I. Human Services %J National Institutes of Health, Common terminology criteria for adverse events (CTCAE) version 4.0. 4 (2009).

[15] Cox JD, Stetz J, Pajak TF. Toxicity criteria of the Radiation Therapy Oncology Group (RTOG) and the European Organization for Research and Treatment of Cancer (EORTC). Int J Radiat Oncol Biol Phys. 1995;31:1341–6. 7713792 10.1016/0360-3016(95)00060-C

[16] Eisenhauer EA, Therasse P, Bogaerts J, Schwartz LH, Sargent D, Ford R, Dancey J, Arbuck S, Gwyther S, Mooney M, Rubinstein L, Shankar L, Dodd L, Kaplan R, Lacombe D, Verweij J. New response evaluation criteria in solid tumours: revised RECIST guideline (version 1.1). Eur J Cancer. 2009;45:228–47. 19097774 10.1016/j.ejca.2008.10.026

[17] Hendry S, Salgado R, Gevaert T, Russell PA, John T, Thapa B, Christie M, van de Vijver K, Estrada MV, Gonzalez-Ericsson PI, Sanders M, Solomon B, Solinas C, Van den Eynden G, Allory Y, Preusser M, Hainfellner J, Pruneri G, Vingiani A, Demaria S, Symmans F, Nuciforo P, Comerma L, Thompson EA, Lakhani S, Kim SR, Schnitt S, Colpaert C, Sotiriou C, Scherer SJ, Ignatiadis M, Badve S, Pierce RH, Viale G, Sirtaine N, Penault-Llorca F, Sugie T, Fineberg S, Paik S, Srinivasan A, Richardson A, Wang Y, Chmielik E, Brock J, Johnson DB, Balko J, Wienert S, Bossuyt V, Michiels S, Ternes N, Burchardi N, Luen SJ, Savas P, Klauschen F, Watson PH, Nelson BH, Criscitiello C, O’Toole S, Larsimont D, de Wind R, Curigliano G, Andre F, Lacroix-Triki M, van de Vijver M, Rojo F, Floris G, Bedri S, Sparano J, Rimm D, Nielsen T, Kos Z, Hewitt S, Singh B, Farshid G, Loibl S, Allison KH, Tung N, Adams S, Willard-Gallo K, Horlings HM, Gandhi L, Moreira A, Hirsch F, Dieci MV, Urbanowicz M, Brcic I, Korski K, Gaire F, Koeppen H, Lo A, Giltnane J, Rebelatto MC, Steele KE, Zha J, Emancipator K, Juco JW, Denkert C, Reis-Filho J, Loi S, Fox SB. Assessing tumor-infiltrating lymphocytes in solid tumors: a practical review for pathologists and proposal for a standardized method from the International Immuno-oncology Biomarkers Working Group: Part 2: TILs in melanoma, gastrointestinal tract carcinomas, non-small cell lung carcinoma and mesothelioma, endometrial and ovarian carcinomas, squamous cell carcinoma of the head and neck, genitourinary carcinomas, and primary brain tumors. Adv Anat Pathol. 2017;24:311–35. 28777143 10.1097/PAP.0000000000000161 PMC5638696

[18] Salgado R, Denkert C, Demaria S, Sirtaine N, Klauschen F, Pruneri G, Wienert S, Van den Eynden G, Baehner F, Penault-Llorca F, Perez E, Thompson E, Symmans W, Richardson A, Brock J, Criscitiello C, Bailey H, Ignatiadis M, Floris G, Sparano J, Kos Z, Nielsen T, Rimm D, Allison K, Reis-Filho J, Loibl S, Sotiriou C, Viale G, Badve S, Adams S, Willard-Gallo K, Loi S. The evaluation of tumor-infiltrating lymphocytes (TILs) in breast cancer: recommendations by an International TILs Working Group 2014. Ann Oncol Off J Eur Soc Med Oncol. 2015;26:259–71. 10.1093/annonc/mdu450 PMC6267863 25214542

[19] Rao U, Lee S, Luo W, Mihm M, Kirkwood J. Presence of tumor-infiltrating lymphocytes and a dominant nodule within primary melanoma are prognostic factors for relapse-free survival of patients with thick (t4) primary melanoma: pathologic analysis of the e1690 and e1694 intergroup trials. Am J Clin Pathol. 2010;133:646–53. 20231618 10.1309/AJCPTXMEFOVYWDA6 PMC3586796

[20] Su L, Chen M, Su H, Dai Y, Chen S, Li J. Postoperative chemoradiotherapy is superior to postoperative chemotherapy alone in squamous cell lung cancer patients with limited N2 lymph node metastasis. BMC Cancer. 2019;19:1023. 31666026 10.1186/s12885-019-6141-z PMC6820909

[21] Fridman WH, Sautès-Fridman C, Galon J. The immune contexture in human tumours: impact on clinical outcome. Nat Rev Cancer. 2012;12:298–306. 22419253 10.1038/nrc3245

[22] Liu JY, Yang GF, Chen FF, Peng CW. Evaluating the prognostic significance of tumor-infiltrating lymphocytes in solid tumor: practice of a standardized method from the International Immuno-Oncology Biomarkers Working Group. Cancer Manag Res. 2019;11:6815–27. 31440080 10.2147/CMAR.S201538 PMC6664256

[23] Donnem T, Hald SM, Paulsen E-E, Richardsen E, Al-Saad S, Kilvaer TK, Brustugun OT, Helland A, Lund-Iversen M, Poehl M, Olsen KE, Ditzel HJ, Hansen O, Al-Shibli K, Kiselev Y, Sandanger TM, Andersen S, Pezzella F, Bremnes RM, Busund L-T. Stromal CD8+ T-cell density: a promising supplement to TNM staging in non-small cell lung cancer. Clin Cancer Res Off J Am Assoc Cancer Res. 2015;21:2635–43. 10.1158/1078-0432.CCR-14-1905 25680376

[24] Donnem T, Kilvaer T, Andersen S, Richardsen E, Paulsen E, Hald S, Al-Saad S, Brustugun OT, Helland A, Lund-Iversen M. Strategies for clinical implementation of TNM-immunoscore in resected nonsmall-cell lung cancer. Ann Oncol. 2016;27:225–32. 26578726 10.1093/annonc/mdv560

[25] Liu H, Zhang T, Ye J, Li H, Huang J, Li X, Wu B, Huang X, Hou J. Tumor-infiltrating lymphocytes predict response to chemotherapy in patients with advance non-small cell lung cancer. Cancer Immunol Immunother. 2012;61:1849–56. 22456757 10.1007/s00262-012-1231-7 PMC11029471

[26] B.W.L. Apar Kishor P. Ganti, Jr., Michael Bassetti, Collin Blakely, et al., Small Cell Lung Cancer, Vison 2. 2022.

[27] Al-Shibli KI, Donnem T, Al-Saad S, Persson M, Bremnes RM, Busund L-T. Prognostic effect of epithelial and stromal lymphocyte infiltration in non-small cell lung cancer. Clin Cancer Res. 2008;14:5220–7. 18698040 10.1158/1078-0432.CCR-08-0133

[28] Horn L, Mansfield AS, Szczęsna A, Havel L, Krzakowski M, Hochmair MJ, Huemer F, Losonczy G, Johnson ML, Nishio M. First-line atezolizumab plus chemotherapy in extensive-stage small-cell lung cancer. N Engl J Med. 2018;379:2220–9. 30280641 10.1056/NEJMoa1809064

[29] Paz-Ares L, Dvorkin M, Chen Y, Reinmuth N, Hotta K, Trukhin D, Statsenko G, Hochmair MJ, Özgüroğlu M, Ji JH. Durvalumab plus platinum–etoposide versus platinum–etoposide in first-line treatment of extensive-stage small-cell lung cancer (CASPIAN): a randomised, controlled, open-label, phase 3 trial. Lancet. 2019;394:1929–39. 31590988 10.1016/S0140-6736(19)32222-6

[30] Park S, Ock C-Y, Kim H, Pereira S, Park S, Ma M, Choi S, Kim S, Shin S, Aum BJ, Paeng K, Yoo D, Cha H, Park S, Suh KJ, Jung HA, Kim SH, Kim YJ, Sun J-M, Chung J-H, Ahn JS, Ahn M-J, Lee JS, Park K, Song SY, Bang Y-J, Choi Y-L, Mok TS, Lee S-H. Artificial intelligence-powered spatial analysis of tumor-infiltrating lymphocytes as complementary biomarker for immune checkpoint inhibition in non-small-cell lung cancer. J Clin Oncol Off J Am Soc Clin Oncol. 2022;40:1916–28. 10.1200/JCO.21.02010 PMC9177249 35271299

